# The Role of Age on Multisensory Bodily Experience: An Experimental Study with a Virtual Reality Full-Body Illusion

**DOI:** 10.1089/cyber.2017.0674

**Published:** 2018-05-01

**Authors:** Silvia Serino, Federica Scarpina, Antonios Dakanalis, Anouk Keizer, Elisa Pedroli, Gianluca Castelnuovo, Alice Chirico, Valentina Catallo, Daniele di Lernia, Giuseppe Riva

**Affiliations:** ^1^ Department of Psychology, Università Cattolica del Sacro Cuore, Milan, Italy.; ^2^ Applied Technology for Neuro-Psychology Lab, IRCCS Istituto Auxologico Italiano, Milan, Italy.; ^3^ Psychology Research Laboratory, IRCCS Istituto Auxologico Italiano, Ospedale San Giuseppe, Piancavallo, Italy.; ^4^ Department of Brain and Behavioral Sciences, University of Pavia, Pavia, Italy.; ^5^ Department of Medicine and Surgery, University of Milano-Bicocca, Monza, Italy.; ^6^ Department of Experimental Psychology, Faculty of Social and Behavioural Sciences, Utrecht University, Utrecht, Netherlands.

**Keywords:** bodily illusion, virtual reality, body image, eating disorders

## Abstract

A growing body of evidence demonstrated that it is feasible to induce ownership over an artificial body to alter bodily experience. However, several uncharted aspects about full-body illusion applications need to be tackled before a complete exploitation of these methods in clinical practice. This work is devoted to explore possible individual age-related differences in shaping changes in body representations induced with a full-body illusion. A total of 40 women were divided into two different age groups according to the median of the variable age. Participants estimated the width of three different body parts (i.e., shoulders, abdomen, and hips) before the entire illusion was induced (baseline), and after the synchronous and the asynchronous conditions. Results revealed that 26-to-55-year-old participants were more resistant to changes induced by the bodily illusion, whereas 19-to-25-year-old participants underestimated their bodies after both conditions. The findings were discussed in terms of the literature exploring age differences in responses to bodily illusion, which could suggest a Bayesian mechanism underlying these individual differences.

## Introduction

In recent years, a growing body of studies has highlighted the potentiality of the “bodily illusions” for altering body representations, namely participants feel significantly fatter or thinner than they really are.^1–3^ A “bodily illusion” can be defined as an experimental setup able to manipulate the experience of being in a body through delivering a synchronized multisensory stimulation (for reviews, see Refs. ^4–7^). One of the most prominent techniques allowing participants to feel that they are the owners of another (whole) body is the *full-body illusion*. The illusory ownership over an artificial body (i.e., a mannequin or a virtual avatar) is achieved by observing from a first-person perspective how the artificial body is being stroked while a synchronous tactile input is perceived on the actual body.^8–11^

For example, when an illusionary embodiment over a virtual body with an enlarged abdomen was induced in young men, they congruently judged themselves to have larger abdomen size.^2^

Evidence deriving from the extant experimental studies for a (a) direct link between perceptual (in terms of a difficulty to provide an accurate estimation of own body size) and affective (in terms of body dissatisfaction) components of body representation disturbances,^3^ and (b) a positive affective response with the full-body illusion modulated by eating disorder psychopathology,^3^ may suggest clinical applications for this method.^12,13^ Indeed, the potentiality of the full-body illusion in decreasing the *overestimation of body size* in patients with anorexia nervosa^14^ and in modulating body representation disturbances in severe and nonoperable obesity patients with regular binge-eating behaviors^15^ was recently reported in the literature.

A recent work analyzing available systematic reviews and meta-analyses about virtual reality (VR) in healthcare clearly indicated the potentiality of this technology not only in eating and weight disorders but also in other clinical conditions, such as anxiety disorders and chronic pain.^16^ As regards eating and weight disorders, VR-based protocols for cue exposure to food stimuli^17,18^ and body image concerns^19^ seem to be effective in reducing eating disorder symptoms. Specifically, it is suggested the exploitation of full-body illusions to further enhance the efficacy of current treatments of body representaton disturbances.^16,20^

However, several uncharted aspects about full-body illusion applications need to be tackled before a complete exploitation of this method in clinical practice. Thus, this work explores possible individual age-related differences in modulating changes of body representation induced with a VR full-body illusion. Indeed, the majority of the aforementioned studies^1,2^ describing changes in body perception (induced by the full-body illusion) were carried out on healthy samples of young women (i.e., mean age of 25 years).

This exploratory study uses a VR full-body illusion^1,14^ to induce illusory ownership over a virtual body with a skinny abdomen in healthy women of different ages to investigate the relationship between age and the magnitude and direction of the changes in body representation.

## Materials and Methods

### Participants

Forty women took part in this study. Participants were recruited through convenience and snowball sampling—in particular, students from the Catholic University of Milan were invited during lessons and asked to refer friends. No economical compensation was given. Inclusion criteria were being female, having between 18 and 55 years of age, having no current or history of neurological illness, and no current physical conditions known to influence body weight/size (for example, pregnancy). In addition, participants were required to have a body mass index (BMI) between 18.5 and 25 kg/m^2^ and not to have a current or history of psychiatric illness (as defined in the DSM-IV-TR, Axis I).^21^

Participants were divided into two different age groups according to the median of the variable “age”: age group 1 (range: 19–25 years old, mean age of 22.55 years [standard deviation, *SD* = 1.82], mean BMI of 21.44 kg/m^2^ [*SD* = 2.06]) and age group 2 (range: 26–55 years old, mean age of 40.20 years [*SD* = 10.64], mean BMI of 21.35 kg/m^2^ [*SD* = 2.16]). The two age groups did not differ in terms of BMI [*t*(38) = 0.121; *p* = 0.904].

### Procedure

All participants provided written informed consent for participating in the study. This study was approved by the Ethics Review Board of the Catholic University of the Sacred Heart (Milan, Italy). At the start of the experiment, participants completed a body size estimation task^14^ by estimating the width of their shoulders, abdomen, and hips (“preillusion estimation”). Subsequently, they were required to wear the HMD-Oculus Rift DK2 to experience the full-body illusion. The virtual body of a woman (∼25 years old, which corresponds to the median age of our sample) with a skinny abdomen (i.e., with a different shape/size in comparison with the actual body of participants) standing upright in a stimulus-free room was used to induce the full-body illusion.^1,14^ The virtual room was developed with the software Unity3D,^a^ whereas the avatar was modeled using the software MakeHuman.^b^

**FIG. 1.**
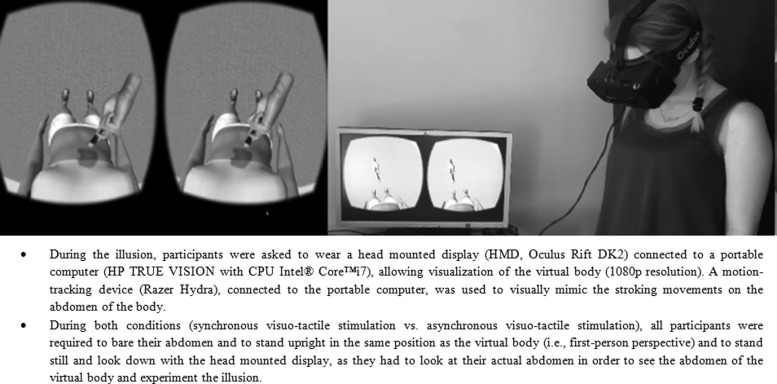
The VR body swap illusion. The illusory ownership over the virtual body (∼25 years old) is achieved by observing from a first-person perspective how the virtual body is being touched on the abdomen while a synchronous input is perceived on the actual body. VR, virtual reality.

The waist circumference of the avatar was 73.95 cm, contrasting the mean waist circumference of both the age group 1 [*t*(19) = −7.078; *p* < 0.001; mean = 84.11; *SD* = 8.34)] and age group 2 [*t*(19) = −7.749; *p* < 0.001; mean = 79.23; *SD* = 10.45)], whereas there was no significant difference in waist circumference between the two groups [*t*(38) = 1.633; *p* = 0.111)]. The full-body illusion (for more details, see Fig. 1) was induced twice after the procedure of previous studies^1,14^: (a) a synchronous visuotactile stimulation (i.e., the experimental condition) and (b) an asynchronous visuotactile stimulation (i.e., the control condition). Both conditions were delivered in a counterbalanced order, following a within-subject design. Both stimulations lasted 90 seconds, a time interval sufficient to induce the illusion as previously reported.^1,14^

In the *synchronous visuotactile stimulation*, the experimenter provided a tactile stimulation on the participants' abdomen for 90 seconds with the brush attached to the motion-tracking device (Razer Hydra). Specifically, there was a synchronous stimulation between the visual input (i.e., a virtual hand holding a brush stroking the abdomen of the virtual body) and what they perceived on their own body. Instead, in the *asynchronous visuotactile stimulation*, there was a delay between the tactile stimulation on the participant's abdomen and the visual input. Indeed, the touch on participants' abdomen was actually recorded by pressing a button on the Razer Hydra at the beginning of the movement. This procedure stopped the image seen by the participants as soon as the touch ended, and then it was replayed in VR when the experimenter finished each touch. After these virtual experiences, participants performed again the body size estimation task (“postillusion estimation”). They also completed the Embodiment Questionnaire, after each illusionary (synchronous/asynchronous) condition.

### Body size estimation task

To investigate whether illusionary ownership over a virtual body with a skinny abdomen would result in changes in body representations between the two groups, participants were required to provide an estimation of the width of three different body parts, namely shoulders, abdomen, and hips. Participants stood in front of a wall and they were asked to estimate the horizontal distance between the left and right side of each body part placing adhesive stickers on the wall. They were explicitly asked to not look at their own body during the task. The body size estimation task was performed three times (before the entire full-body illusion, after the synchronous visuotactile stimulation, after the asynchronous visuotactile stimulation). The actual width of the body parts was measured at the end of the experiment.

### Embodiment Questionnaire

A short (15-item) Embodiment Questionnaire,^1,22^ rated on a 7 point Likert scale (ranging from 1, *fully disagree* to 7, *fully agree*), was administered after completing each condition (i.e., synchronous vs. asynchronous visuotactile stimulation) to assess the extent to which participants experienced the full-body illusion on three different components. The first one measured the *body ownership* (e.g., “I felt as the virtual body was my body”); the second, the *self-location* (e.g., “I felt as I was inside the virtual body”); and the third, the *sense of agency* (e.g., “I had the feeling that I had the control over the virtual body”). The three components were obtained by calculating the mean scores from the items.

## Results

### Embodiment Questionnaire

A mixed analysis of variance (ANOVA) with group and condition was carried out for each of the three subscales of the Embodiment Questionnaire (i.e., ownership, self-location, and sense of agency). Concerning ownership, a main effect of *condition* emerged (Table 1), with significantly higher scores in the synchronous condition than in the asynchronous condition (*p* = 0.043). A same pattern of results emerged for self-location, with higher scores in the synchronous condition than in the asynchronous condition (*p* = 0.002). Concerning sense of agency, no significant differences were found between the two conditions. Neither a main effect of group nor interactions between group and condition were found (see Table 1 for full statistics) for the three measures (i.e., ownership, self-location, and agency), suggesting the absence of any age-related effect.

**Table 1.** Results Obtained from the Embodiment Questionnaire

		*Age group 1*	*Age group 2*		F	p	*Partial η^2^*
Ownership	Synchronous condition	3.57 (1.12)	3.78 (1.41)	Group	0.001	0.993	0.001
	Asynchronous condition	3.48 (1.09)	3.28 (1.52)	Condition	4.363	0.043	0.103
				Condition × group	1.963	0.169	0.049
Self-location	Synchronous condition	4.32 (1.00)	4.46 (1.47)	Group	0.090	0.766	0.002
	Asynchronous condition	3.72 (1.32)	3.82 (1.65)	Condition	11.499	0.002	0.232
				Condition × group	0.011	0.919	0.001
Agency	Synchronous condition	4.00 (1.74)	3.40 (2.02)	Group	0.748	0.393	0.019
	Asynchronous condition	3.72 (1.63)	3.37 (1.94)	Condition	0.616	0.437	0.016
				Condition × group	0.428	0.517	0.011

Data are shown as mean (*SD*). For all analyses, df = 1, 38.

*SD*, standard deviation.

### Body size estimation task

We computed the percentage of misestimation for each participant and for each body part, following Keizer et al.'s procedure^14^: *percentage of misestimation* = (estimated size−actual size)/actual size) × 100. Specifically, a negative value represented an underestimation, whereas a positive value represented an overestimation. Independent sample *t*-test indicated that there were no significant difference among the two groups in body size estimations at baseline [*t*_shoulders_(38) = 0.205; *p* = 0.838; *t*_abdomen_(38) = −1.615; *p* = 0.115; *t*_hips_(38) = −1.845; *p* = 0.073]. According to a mixed ANOVA relative to the shoulders width estimation, no main effect of *condition* or *group* emerged. Importantly, the interaction between *condition* and *group* was significant (Table 2). Bonferroni-corrected pairwise comparisons revealed that 19-to-25-year-old participants significantly underestimated shoulders width in the synchronous postillusion condition compared with the preillusion–baseline phase (*p* = 0.05) (Table 3). No other comparisons resulted significant.

**Table 2.** Effect of Body Swap Illusion in Inducing Changes in Body Size Estimation

	*Age group 1*	*Age group 2*		F	p	*Partial η^2^*
Shoulders						
Preillusion size estimation	−7.663 (15.523)	−8.715 (16.875)	Group	1.363	0.250	0.035
Synchronous postillusion size estimation	−15.132 (15.225)	−4.332 (17.124)	Condition^a^	2.036	1.38	0.051
Asynchronous postillusion size estimation	−15.014 (14.700)	−9.802 (13.644)	Condition × group^b^	3.927	0.024	0.094
Abdomen						
Preillusion size estimation	−9.931 (23.838)	2.338 (24.208)	Group	4.996	0.031	0.116
Synchronous postillusion size estimation	−14.909 (23.484)	2.365 (25.395)	Condition^a^	1.081	0.344	0.028
Asynchronous postillusion size estimation	−16.768 (21.828)	2.237 (28.161)	Condition × group^b^	1.105	0.337	0.028
Hips						
Preillusion size estimation	0.314 (19.168)	11.836 (20.305)	Group	8.902	0.005	0.190
Synchronous postillusion size estimation	−3.877 (19.893)	18.152 (26.355)	Condition^a^	1.660	0.197	0.042
Asynchronous postillusion size estimation	−6.666 (18.047)	13.238 (18.048)	Condition × group^b^	3.237	0.045	0.078

Data are shown as mean (*SD*).

^a^ df = 1, 38.

^b^ df = 2, 76.

**Table 3.** Interaction Between Condition and Group for Shoulders Estimates

	*Mean difference (I*−*J)*	*Standard error*	p
Age group 1			
Preillusion size estimation			
Synchronous	7.469	2.978	0.050
Asynchronous	7.351	3.082	0.066
Synchronous postillusion size estimation			
Preillusion	−7.469	2.978	0.050
Asynchronous	−0.118	2.915	1
Asynchronous postillusion size estimation			
Preillusion	−7.351	3.082	0.066
Synchronous	0.118	2.915	1
Age group 2			
Preillusion size estimation			
Synchronous	−4.383	2.978	0.448
Asynchronous	1.087	3.082	1
Synchronous postillusion size estimation			
Preillusion	4.384	2.978	0.448
Asynchronous	5.471	2.915	0.205
Asynchronous postillusion size estimation			
Preillusion	−1.087	3.082	1
Synchronous	−5.471	2.915	0.205

Bonferroni pairwise comparisons.

Referring to the abdomen width estimation, a main effect of *group* emerged, but not of *condition* neither a significant interaction (Table 2). This means that, regardless of condition, 19-to-25-year-old participants (estimates mean = −13.869; standard error = 5.134) reported a larger percentage of misestimation for abdomen, underestimating its size, in comparison with 26-to-55-year-old participants (estimates mean = 2.359; standard error = 5.134). Finally, about hips, a main effect of *group* and a significant interaction between *condition* and *group* (Table 2) emerged. Participants of 26-to-55-year old showed again a larger percentage of misestimation (estimates mean = 14.409; standard error = 4.223) in comparison with 19-to-25-year-old participants (estimates mean = −3.410; standard error = 4.223). Interestingly, Bonferroni-corrected pairwise comparisons revealed that 19-to-25-year-old participants' misestimation of hips width significantly increases from preillusion to asynchronous postillusion (*p* = 0.039) (Table 4). Although 26-to-55-year-old participants showed a tendency to overestimate, 19-to-25-year-old participants exhibited the opposite tendency (i.e., the underestimation), which increased after the illusion. No other comparisons were significant.

**Table 4.** Interaction Between Condition and Group for Hips Estimates

	*Mean difference (I*−*J)*	*Standard error*	p
Age group 1			
Preillusion size estimation			
Synchronous	4.191	3.290	0.632
Asynchronous	6.980	2.674	0.039
Synchronous postillusion size estimation			
Preillusion	−4.191	3.290	0.632
Asynchronous	2.789	3.260	1
Asynchronous postillusion size estimation			
Preillusion	−6.980	2.674	0.039
Synchronous	−2.789	3.260	1
Age group 2			
Preillusion size estimation			
Synchronous	−6.316	3.290	0.187
Asynchronous	−1.402	2.674	1
Synchronous postillusion size estimation			
Preillusion	6.316	3.290	0.187
Asynchronous	4.915	3.260	0.419
Asynchronous postillusion size estimation			
Preillusion	1.402	2.673	1
Synchronous	−4.915	3.260	0.420

Bonferroni pairwise comparisons.

## Discussion

The aim of this study was to explore possible age-related differences in body representation changes after a VR full-body illusion. Results from the Embodiment Questionnaire^1,22^ indicated that the feeling of owning a virtual body and being in the same place of the virtual body were higher in the synchronous condition for all participants. This means that, regardless of age, participants reported to experience the illusion in terms of body ownership and self-location, but not in terms of agency, as expected since the avatar was static. Our findings revealed that, independently of the type of stimulation, 19-to-25-year-old participants globally reported an increase of underestimation after embodying a virtual body with a skinny abdomen (especially for shoulders and hips), whereas 26-to-55-year-old participants appeared more resistant to changes of their body perception after the bodily illusion.

To date, very few studies, with contrasting results, have explored possible age-related differences in responses to the bodily illusions. Some experimental studies using the “rubber hand illusion” (RHI) (i.e., the prototypical paradigm of the class of bodily illusions, full-body illusion included^23^) have reported that children show a larger proprioceptive drift toward the fake hand in comparisons with young adults,^24,25^ but this difference is not mirrored in their explicit feeling of ownership.^24^ Kállai et al.^26^ recently found that old participants (mean age: 65.9 years old) reported less vivid ownership toward the rubber hand compared with younger participants (mean age: 27.7 years old).

A very recent study carried out by Palomo et al.^27^ did not find any differences among three different target groups (20–35 years old, 36–60 years old, and 61–80 years old) in response to the RHI, suggesting that this illusion elicits the same “embodiment process” across different ages. Two studies specifically focused on middle-aged participants, that is, a target group similar to our study. Tajadura-Jiménez et al.^28^ used the so-called enfacement illusion, that is, the delivery of a synchronous multisensory stimulation between one's own face and another person's face to induce changes in self-identification. They found that middle-age participants were more resistant to the illusion than younger participants; it might be that the huge changes in visual body appearance in the middle age require a larger plasticity of body representation.

Using a variant of the RHI, Graham et al.^29^ found a decrease in the perception of the illusion with age. These authors introduced a Bayesian mechanism to explain how illusion works: it is possible to experience two spatially congruent perceptions as linked thanks to prior probabilities associated with one's own history of perceptual experiences. This results in the illusory perception of the “fake” hand as one's own hand. With increasing age, individuals have a great amount of experience of spatially congruent perceptions, thus it might decrease the prior probabilities of two spatially incongruent perceptions as originating from one's own hand, reducing the possibility of experiencing the illusion. Both these two interpretations can be embraced to explain the lower level of plasticity of 26-to-55-year-old participants' body representation. Piryankova et al.^22^ reported that their participants changed the “experienced body” (in their terminology, “the body that the participant feels he/she has at that moment”) before any type of stimulation, but only when they viewed an underweighted (and not an overweighted) virtual body. They discussed these findings arguing that, beyond the role of visual input from seeing the virtual body, participants probably used the “memory” of their own body^30–33^ for estimating the physical body dimensions.

Accordingly, in our experiment, the illusion might affect body perception in 19-to-25-year-old participants, but not that of the 26-to-55-year-old participants, since their body representation (stored in memory^33^) was more stable. During aging, there is a shift in cortical responses from sensory regions to executive regions (the so-called compensation hypothesis^34^). According to a Bayesian approach,^35–37^ the relative “lower plasticity” of the body representation in 26-to-55-year-old participants could be interpreted as a more rigid use of predictive strategies acquired during the lifespan (as observed also by Graham et al.^29^) instead of adapting these strategies to sensory inputs.^38^ However, future research to support this suggestion is required.

It is crucial to note that the difference between the two groups emerged independently from the conditions, thus suggesting that solely viewing a virtual body while it was stimulating can modify the body perception for 19-to-25-year-old participants. It was likely that participants experienced this illusion also in the asynchronous condition, even at less extent. Indeed, it has already been demonstrated that a first-person perspective (i.e., an avatar being spatially coincident with the position of the participant) of a realistic virtual body substituting participants' own body is sufficient to generate an illusory feeling of ownership and changes in body representations,^1^ even though after an asynchronous stimulation.^1,10,11^

In conclusion, this study represents the first attempt to provide evidence about the effect of age on multisensory bodily experience offering valuable insights to guide and stimulate future research in this area. In particular, it may stimulate research investigating possible clinical application of bodily illusions^13,16^ for shaping body perception also in eating disorders among women in midlife.^39^

However, despite these promising results, some limitations arose. First, it should be noted that we primarily opted for only a self-report measure of embodiment, although it would have of interest to have also physiological (i.e., skin conductance response or body temperature) measurements. Second, future studies should investigate the effect of different ages, ranging from 18-year-olds to elderly individuals, as in the study of Palomo et al.^27^ Third, it should be acknowledged that our sample counted only female participants, thus current findings cannot be spread also to male population. Finally, it should be noted that the second group included a larger life span period—women older than 35 years—with a reduced probability of pregnancy.^40^

Future studies should use different avatars of different ages and investigate the role of psychological factors (i.e., body esteem and self-esteem) and include also the use of simulation/stimulation technologies able to modulate the inner experience of the body^41^ to fully capture the complicated patterns of age and body representations across lifespan.

## Notes

a. www.unity3d.com

b. www.makehuman.org/
